# Microscopic Characterization of Biological and Inert Particles Associated with Spacecraft Assembly Cleanroom

**DOI:** 10.1038/s41598-019-50782-0

**Published:** 2019-10-03

**Authors:** Ganesh Babu Malli Mohan, Moogega Cooper Stricker, Kasthuri Venkateswaran

**Affiliations:** 1 Jet Propulsion Laboratory, California Institute of Technology, Biotechnology and Planetary Protection Group, Pasadena, CA 91109 USA; 2 Present Address: Center for Research in Infectious Diseases, School of Chemical and Biotechnology, SASTRA Deemed to be University, Thanjavur, 613 401 India

**Keywords:** Applied microbiology, Environmental impact

## Abstract

NASA cleanrooms are certified by particle counts and are humidity-controlled, temperature-regulated, and oligotrophic in nature for assembling spacecraft subsystems. Microorganisms, which are not part of the cleanroom certification metrics, should not be overlooked when assessing the cleanliness of the facility since they can enter through soil or air, shed from humans, adapt to the oligotrophic conditions, and subsequently could contaminate spacecraft. These biogenic particles need to be identified to extend our knowledge of biological contamination for future NASA mission use. This study collected particles from the cleanroom and estimated the distribution of fallout microbial cell and inert dust particles using microscopy and molecular techniques. Aluminum coupon-based polycarbonate filter assemblies were deployed in the spacecraft assembly cleanroom facility to collect fallout particles. Epifluorescence and electron microscopy showed that particles varied in size and structure, and displayed live/dead biological and inert particle signatures from sources that include spores and fungal hyphae. Additionally, correlative epifluorescence and field emission scanning electron microscopy, combined with energy-dispersive X-ray analysis (for elemental compositions) methods, differentiated whether microbes adhering to particles were live/dead cells or inert particles. This visualization approach allowed for the classification of microorganisms as being standalone (free-living) or associated with a particle, as well as its characteristic size. Furthermore, time-course microscopy was used to determine the microbial cell growth and confirm the biological/molecular identification. Routine investigation of cleanroom biological and inert fallout particles will help to determine the biological load of spacecraft components and will also have direct relevance to the pharmaceutical and medical industries. One of the main objectives for NASA’s current and future missions is to prevent forward and back contamination of exploring planets. The goal of this study is to determine the association of microorganisms with the inert, natural cleanroom fallout particles and to ascertain whether microorganisms prefer to adhere to a particle size. A novel microscopy technique was developed, and by utilizing various molecular techniques, particles and associated microbial phylogeny were characterized. An accurate assessment of the microbes associated with cleanroom particles is necessary to protect the health of the people who occupy the room for long duration for aeronautical, medical, and pharmaceutical industries.

## Introduction

Microorganism(s) associated with fallout particles are a critical concern for fabrication of sensitive products with highly liable surface particulate contamination, including medical devices, pharmaceutical products, electronic supplies, and spacecraft assembly^1–7^. Fallout particles vary greatly in size (ranging from 0.1 μm to 1000 μm), morphology, and elemental composition, and can originate from sources such as human, cosmetics, cargo, and particles transported in the cleanroom environment from the external environment, which varies by geographic location^8^. Therefore, it is necessary to understand the size distribution and chemical composition of natural fallout particles in critical indoor environments such as intensive care units (ICUs), spacecraft assembly facilities, and other industrial cleanrooms^7,9^. However, reports on the biological burden associated with cleanroom natural fallout particles are not available. Even though National Aeronautics and Space Administration (NASA) cleanrooms have been maintained with controlled airflow circulation, temperature, humidity, and rigorous cleaning, they are not sterile^10–12^.

Since Viking, NASA has been assembling unmanned robotic spacecraft in cleanroom environments and continuously monitoring microorganisms to mitigate the forward contamination of other planets and bodies in the solar system^10,13–15^. Since 2000, in addition to the NASA spore assay, advanced next-generation sequencing methods such as targeted gene(s)^16,17^, as well as DNA microarray and shotgun metagenome sequencing^18^, were carried out to identify the microbial burden and their distribution on the cleanroom microbial ecosystem^19^, but no studies on the characterization of biological materials associated with fallout particles have been completed. In the past, particulate and microbial contamination were not assessed in tandem but analyzed separately using two distinct sets of witness coupons^7,20^. Therefore, research needs to be conducted to estimate the distribution of microbial communities and their association with cleanroom particles from the same set of materials.

Large sets of science data were available on the indoor/outdoor airborne microbes from bulk materials that employed traditional culture-dependent and molecular approaches to identify the microbial diversity in environments specific to cleanrooms^15,18,21^. In addition, visual characterization of particles were commonly captured using epifluorescence and field emission scanning electron microscopy (FESEM) with energy-dispersive X-ray analysis (EDXA) since these approaches provide information on both morphology and organic elemental composition^22^. However, electron microscopy is limited to visualizing only the morphology of the target and cannot be used to discern biological particles from individual inert particles. In this communication, “inert particle” is defined as the particle that did not show any carbon signature when EDXA analysis was performed. Few studies utilized both microscopy and molecular biological techniques to concurrently link the microorganism to the environmental dust particles and determine whether microorganisms were associated with particles or were free-living^23,24^. Recent advances in microscopy techniques such as correlative light and electron microscopy procedures have been utilized to image the same particles in the same sample with two different microscopy^25^. This integrated novel approach will help to understand whether the particles in question are biological or inert particle by characterizing morphology (microscopy), chemistry (EDXA), and biology (growth) of the contaminants. Particles were collected in multiple NASA cleanrooms, including the Jet Propulsion Laboratory (JPL) assembly facility cleanrooms, using various microbiology and molecular biology techniques^6,26–28^. This is the first time a study was systematically conducted to characterize microorganisms of NASA spacecraft cleanroom particles and its direct association with inert fallout particles.

Previously, we devised a witness coupon to collect fallout particles using polycarbonate (PC) filters and confirmed the presence of microbial cells using epifluorescence imaging and ImageJ analysis^29^. This study has applied the lessons learned from the prior study on an entirely new set of deployed coupons with the goal of characterizing microbial association with particles and its distribution profile in International Organization for Standardization (ISO) 5 and ISO 7 spacecraft assembly cleanroom environments. The main objective of this study is to develop a correlative epifluorescence microscopy (EFM) based technique combined with FESEM-EDXA (cEFM-FESEM-EDXA) to acquire information on the same field of image on the viability (live/dead) of microbial cell, morphology of the structure, and elemental composition of the fallout particle. Furthermore, microorganisms associated with particles were isolated and identified.

## Materials and Methods

### Particulate characteristics of JPL spacecraft assembly facility cleanrooms

The JPL spacecraft assembly facility examined in this study is maintained with cleaning regimens at frequencies appropriate to the current level of activity of the cleanroom facility. At the time of the sampling events, a significant amount of assembly activity of critical spacecraft hardware occupied the clean room. Thus, cleaning frequency for facility at the time of sampling was daily. Daily cleaning regimens of cleanroom facility maintenance include the replacing of tacky mats, wiping surfaces, and vacuuming/mopping floors using clean room-certified sanitizing agents (disinfectants, alcohol, or ultrapure water). All personnel who enter the cleanrooms must take appropriate actions to minimize the influx of particulate matter. Specific entry procedures varied depending on the certification level of the clean room and the presence or absence of mission hardware. General precautions include donning of cleanroom-certified garments to minimize exposure of skin, hair, and the regular clothing of the personnel. General precautions also include prohibition of the usage of cosmetics, fragrances, body spray, and hair gels before entry into the cleanroom. The air of both facilities was filtered through high-efficiency particle air (HEPA) filters. The total number of particles of the size >0.5 µm were <10,000/ft^3^ of air during the period of this study even though this facility certification is ISO-7.

### Fallout particles collection and processing

**Figure 1:**
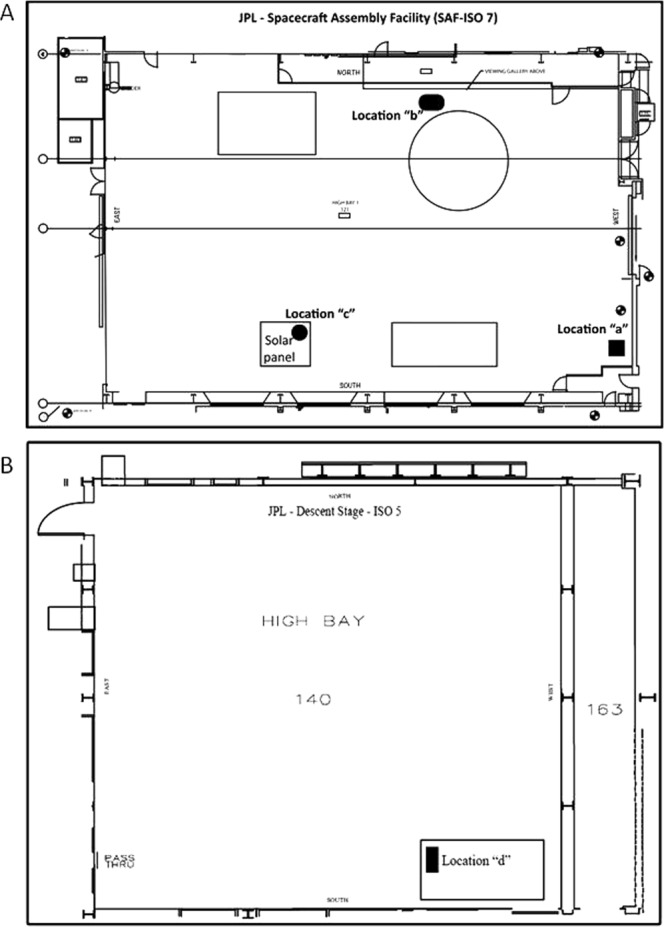
Biological fallout coupon deployed location at JPL clean room test bed. (**A**) 2D line illustration of JPL-Spacecraft Assembly facility (ISO – 7). Four consecutive fallout coupons were deployed for every two weeks intervals. Locations highlighted in various shapes represented deployed coupons. Square shaped - location “a”; rod shaped - location “b” and circle shaped - location “c”. (**B**) 2D illustration of JPL-Decent Stage-233(ISO – 5). Rectangle highlights locations that coupons were deployed – location “d”.

An aluminum frame was assembled as described in Malli Mohan *et al*.^29^ to secure sterile 0.2-µm pore size polycarbonate (PC) membrane filters. These witness coupons were deployed to trap fallout particles and replaced every two weeks for four consecutive sampling periods in the JPL spacecraft assembly facility cleanrooms (ISO 5 and ISO 7; Fig. 1). Coupons were removed and carefully transferred to the lab, and the PC filters aseptically disassembled from the coupons and processed. Next, the PC filters and the trapped particles were mounted on to the borosilicate glass Buchner filter funnel device (Millipore, USA), washed with sterile water, and stained with BacLight stain mixture. The stain mixture contained equal volumes of propidium iodide (PI) and SYTO 9 solution provided in the BacLight viability kit (catalog no. L7012; Molecular probes, Invitrogen, Carlsbad, USA). This step was followed by a 15-min incubation at room temperature in the dark, and then the PC filters were washed four times with particle-free water.

### Fluorescence microscopy

BacLight-stained PC-filter images were acquired by an Axioplane microscope equipped with an AxioCam camera (Carl Zeiss, CA). The entire PC filter was imaged and reconstructed by the MosaiX function in the AxioVision software (Carl Zeiss, CA). These images were further processed to determine the particle distribution with the ImageJ program^30^.

### FESEM-EDXA imaging

The PC filter was aseptically detached from the aluminum frame and mounted on carbon stubs for carbon coating. A 5-nm thin film of carbon was deposited on the surface of each PC filter using a vacuum coating unit (Leica EM ACE600, USA). Samples were observed under high vacuum at 5–20 kV, with a 6–8 mm working distance using the secondary electron (SE) detector mode on the FEI Sirion scanning electron microscope (FESEM Quanta 200 FEI, OR, USA). Electron microscopy images were recorded at magnifications ranging from 100x to 20,000x. To examine the particle chemical composition, EDX analysis was carried out at each field using a Phoenix EDXA tower, and the spectra were captured using Genesis software.

### Validation of time-course microscopy

Time-lapse microscopy was validated using a known microorganism before being used to characterize fallout particles. *Bacillus pumillus* SAFR032 spores were purified as per established protocol^31^. Appropriate aliquots of the spore suspensions were trapped onto PC filters (*n* = 6), carefully placed on to the trypticase soy agar (TSA) plate, incubated for 180 mins at 30 °C, and every 30 mins, one PC filter each (0, 30, 60, 90, 120, 180 min) was stained with BacLight and observed under epifluorescence microscopy^32^.

### Isolation and identification of microorganisms associated with fallout particles

Microorganisms associated with the fallout particles trapped on PC filters (*n* = 2) were isolated by placing the PC filters onto Reasoner’s 2 A (R2A) agar and incubated at 30 °C for 16 hrs. One of these pre-enrichment treated PC filters was transferred into a fresh R2A agar via replica-plating^33^ and incubated at 30 °C for 5 days, which provided enough time to form well-developed microbial colonies. The other PC filter was used for the EFM method to count viable cells after BacLight staining as described above. The microbial colonies developed on R2A plates were subsequently picked and archived in the semisolid R2A slants (agar media diluted 1:10) for further study. Once a culture was confirmed to be pure, the UltraClean DNA kit (MO Bio, Carlsbad, CA) was used to extract the DNA. Next, polymerase chain reaction (PCR) was performed to amplify the 1.5 kb 16S rRNA gene to identify bacterial strains using the forward primer, 27 F (5′-AGA GTT TGA TCC TGG CTC AG-3′) and the reverse primer, 1492 R (5′-GGT TAC CTT GTT ACG ACT T-3′)^34,35^. The PCR conditions were as follows: denaturation at 95 °C for 5 mins, followed by 35 cycles consisting of denaturation at 95 °C for 50 sec, annealing at 55 °C for 50 sec, extension at 72 °C for 90 sec, and finalized by extension at 72 °C for 10 mins. The sequences were assembled using SeqMan Pro from the DNAStar Lasergene Package (DNASTAR Inc., Madison, WI). The bacterial sequences were searched against EzTaxon-e database. The identification was based on the closest percentage similarity (>97%) to previously identified microbial type strains. The 16S rRNA sequences of the isolates were deposited in the NCBI GenBank under accession no.: MN006145 – MN006154.

### Determination of cleanroom particle size using ImageJ

Automated ImageJ commands were carried out to count the particles from the acquired EFM images. First, the following command was used to open an image for a particle analysis using ImageJ: Image > Adjust > Threshold > Max Entropy > Apply. Next, the split channels option was used to split the red and green fluorescence particles: Image > Color > Split Channels; and for color threshold: Image > Adjust > Threshold. Finally, the particles were counted using the commands: Analyze > Analyze Particles, with the upper and lower limits of the particle size set at 0 to infinity, selected to “show outlines” and checked the box to “Summarize.” Each counted particle was outlined and numbered with the diameter of the particle^30^.

The ratio of microorganisms to particles was determined using a multi-step process. First, the sizes of each detected particle were determined as described above. These particles were binned as inert (red fluorescence) or viable/intact particles (green fluorescence). Second, images of the fallout particles were captured using FESEM to determine the associated particle size, and its chemical composition was confirmed using EDXA. The shortfall of the EFM method alone is that it does not give ample resolution to determine whether the green fluorescence comes from one or more closely packed particles. The use of FESEM eliminated this deficiency and allowed the study to determine the fraction of particles that are multiples for each particle size. The FESEM observations further informed the bin size selection as displayed in Supplementary Table S1. The ratio of microorganisms to particles was enumerated using the combination of inert/viable particles by size per the EFM and the fraction of singlet/multiple particle combinations as gathered by FESEM.

## Results

### Validation of the PC filter for collecting cleanroom particles

**Figure 2:**
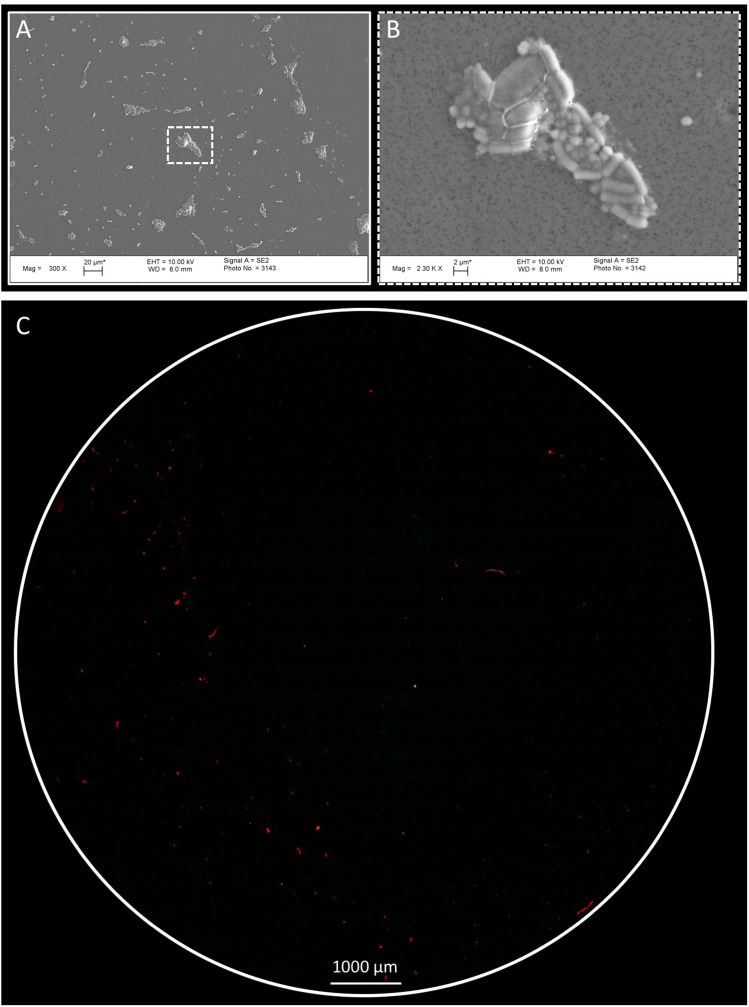
Epi-fluorescence imaging of fallout particles using BacLight staining. (**A**) Model microbial community were filter on PC filter, followed by carbon coated and visualized by FE-SEM (x300). (**B**) Higher magnification (x23000) of typical FE-SEM image of white dotted boxed region shown in (**A**). (**C**) Aluminum frame PC filter witness coupons were assembled in aseptic condition with sterile polycarbonate filters and deployed at JPL-SAF. After two weeks, the coupons were collected, PC filter aseptically removed, followed by staining with SYTO-9 and Propidium iodide (PI) and washed twice with PBS. Shown is an example of typical epi-fluorescence microscope image of fallout particles observed on entire PC filter. Red pseudo color stained by PI and Green.

The PC filter was tested initially using BacLight staining EFM in order to understand the autofluorescence of the filters. A model microbial community (MMC) inoculum^36^ was spiked onto the PC filters, and control PC filters were used to measure clean backgrounds (Fig. S1A). Not surprisingly, MMC trapped on PC filters were appropriately stained with no autofluorescence and low background noise (Fig. S1B). Moreover, intact morphologies of MMC were captured under FESEM without sample processing (e.g., glutaraldehyde fixation), which confirmed that the PC filters were appropriate for trapping particles and acquiring microscopy images of biologicals and inert dust particles associated with the cleanroom particles (Fig. 2A,B). Since there was no background noise in the control PC filters (Fig. S1A), the biogenic origins of the particles (biologicals or inert particles) collected in the PC filters were determined by BacLight staining. In addition, a closer observation of the EFM images indicated that fallout particles varied in size and showed clear, distinct morphology.

### Collection of cleanroom particles

**Figure 3:**
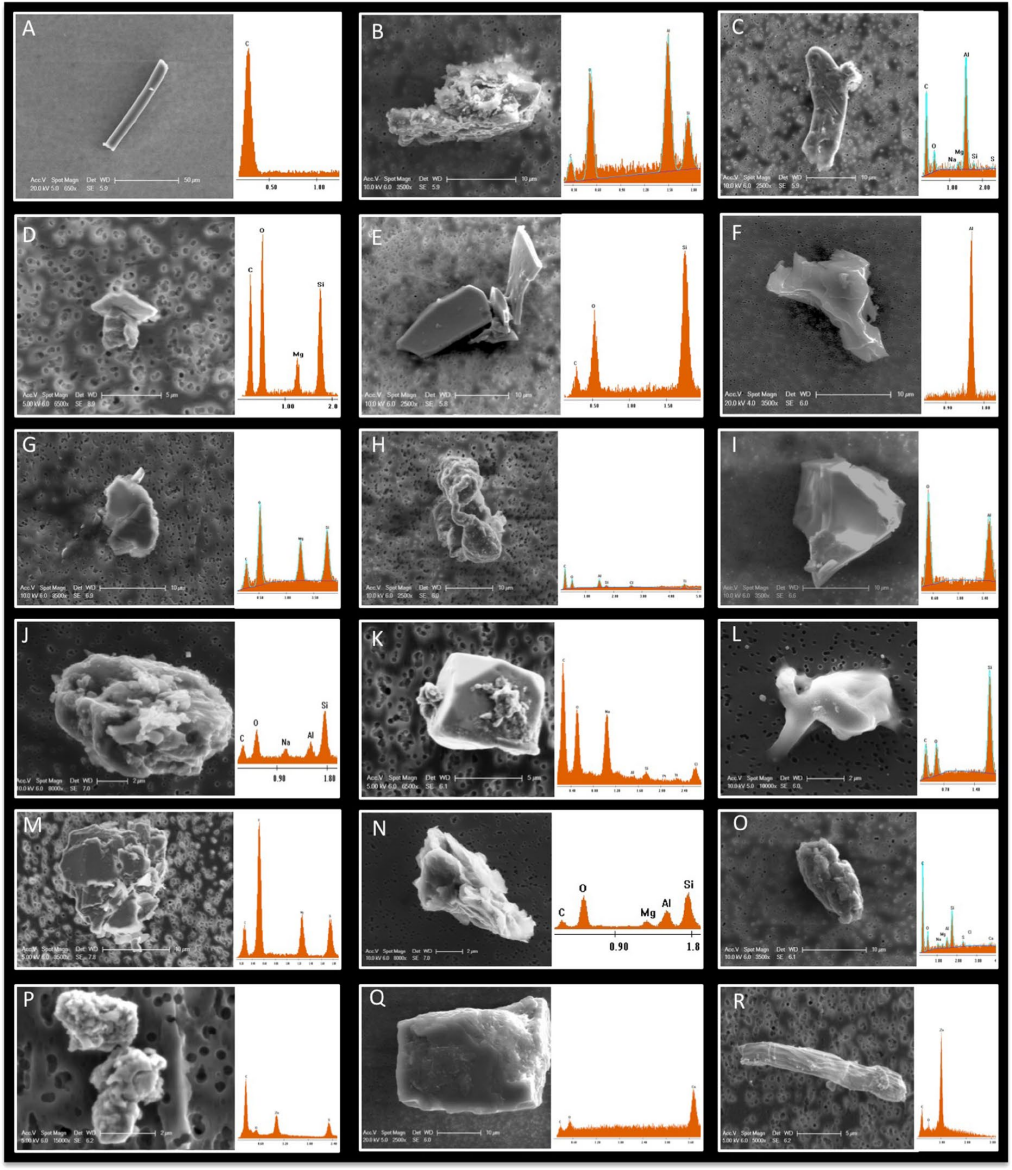
FESEM-EDXA photomicrographs of fallout particles from JPL-cleanroom. Fallout coupons collected from JPL cleanroom were carbon coated, visualized by FESEM, and elemental composition analyzed by Energy Dispersive X-ray Analysis EDXA. (**A**) A tubular structure of pure carbonaceous fiber (likely to be nylon or carbon fiber) with “c” signature only; (**B**) Aluminosilicate with irregular shape; (**C**) An irregular, rod shaped aluminum rich particle with carbonaceous element; (**D**) An irregular magnesium silicate particle with carbonaceous element; (**E**) Fly like shaped crystalline silica; (**F**) Aluminum rich particle with irregular shape; (**G)** Magnesium silicate particle with oval shape; (**H**) Carbonaceous rich aluminosilicate, irregular rod tubular with little titanium anthropogenic particle; (**I**) A triangular shaped Aluminum oxide particle; (**J**) Na-feldspar particle; (**K**) Carbonaceous particle associated with a polygon shaped Na-feldspar particle; (**L**) Quartz with irregular shape; (**M**) Magnesium silicate with irregular shape; (**N**) Pyrope (Mg-Al-Si-O) with irregular triangular shape; (**O**) Carbonaceous rich Ca-Magnesium aluminosilicate with rough rod shape (Likely to be of biological origin); (**P**) Two irregular rod shaped Zinc sulfide particles associated with carbonaceous particles (likely to be a bacterial cell membrane covered with Zinc particles); (**Q**) Calcium rich particle with rectangular morphology; and (**R**) Irregular tubular Zinc rich particle. The displayed images are fallout dust particle with size ranges between 2–75 µm, respectively.

A total of 24 PC filters were exposed to an ISO 7 environment, and 8 PC filters were exposed to an ISO 5 environment. Fallout particles were collected after a two-week duration for four consecutive months in JPL cleanrooms. The FESEM microscopy analysis of the PC filters revealed various sizes and shapes of the particles, but EDX chemical analysis was required to differentiate biological materials (presence of elemental carbon) from inert particles (Fig. 3). By employing this approach, a total of 300 individual or aggregate particles were captured to determine the biological particles (Figs 3, S2 and S3).

A closer observation of an individual fallout particles revealed several morphological structures that include irregular shapes (Fig. 3B,D,F,H,L,P), such as rectangular, triangular, tubular/rod, polygon, oval, and distinct miniature fly-like structures (Fig. 3E). Chemically, these fallout particles were composed of C, O, Mg, Na, Zn, Al, Si, S, Cl, Ca, Ti, and Fe, in varying abundances and categorized as biogenic, geogenic, or anthropogenic particles^22^. Geogenic particles, such as quartz (Si-O) (Fig. 3E,L), aluminosilicates as Na-feldspar (Fig. 3J), or fly ash (Fig. 3B) were comprised of minor amounts of C signature, suggesting that these particles were carbonaceous particles and inorganic materials. Furthermore, single carbon tubular particles were dominated with a 100% weight of C element, which is an anthropogenic particle, while aluminum metallic particles showed a 100% weight of Al element. Notably, particles rich in sodium chloride and sulphite exhibited regular shapes, like pentagon structure as illustrated in Fig. 3K.

**Figure 4:**
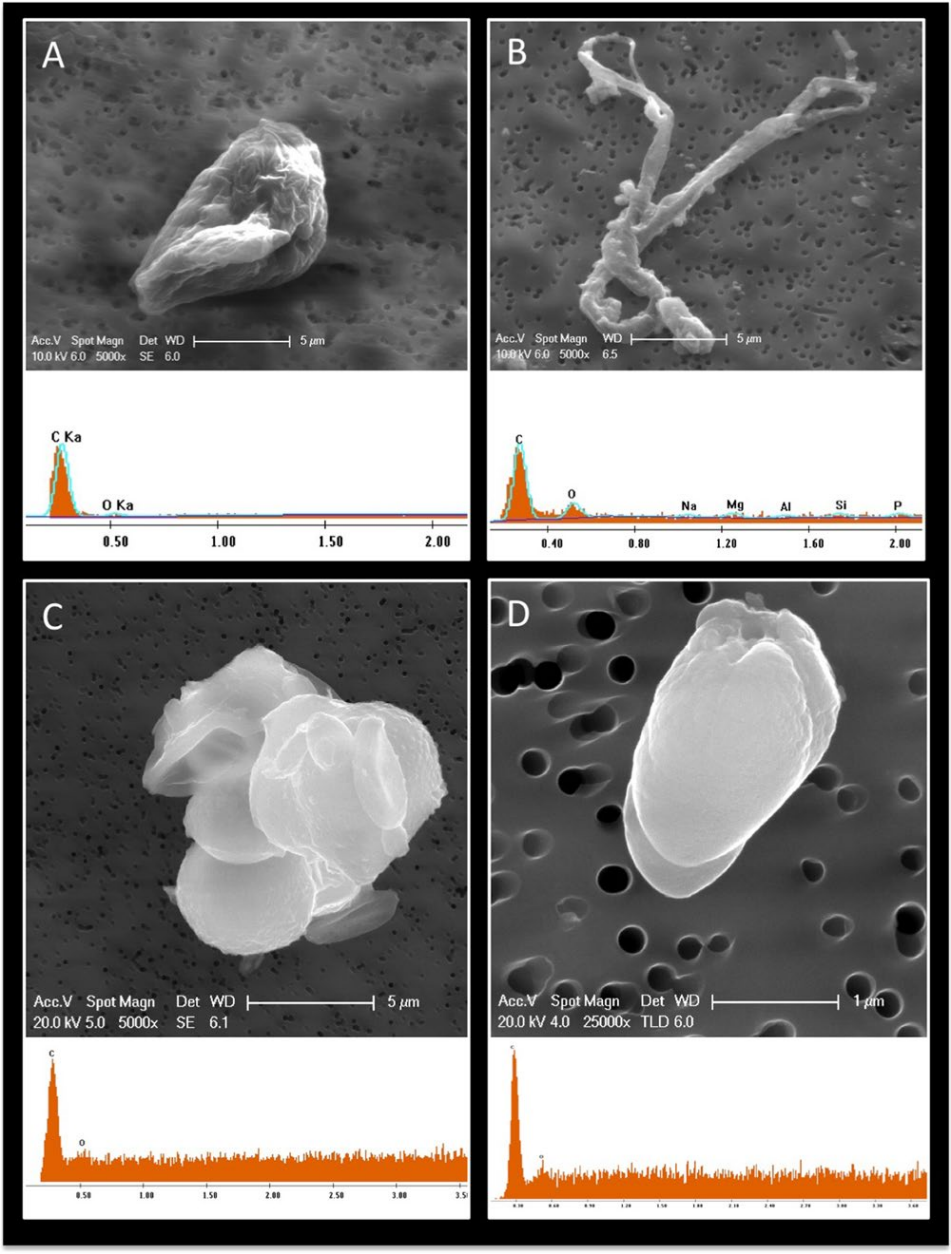
Visualization of biological fallout particles using FESEM coupled with EDAX. Fallout coupons collected from JPL cleanroom were carbon coated, visualized by FESEM, and analyzed for elemental composition by EDAX. (**A**) Image (x5000) of a biological particle that seems likely to be a pollen or spore; (**B**) Image (x5000) of a fungal hyphae; (**C**) Image (x5000) of a biological particle that seems likely to be fungal spore aggregates or a budding yeast event is highlight by arrow; and **(D)** Image (x25000) of bacterial particles that seem likely to be bacterial spores. (**A**–**D**) EDAX spectral of shows “c” and “o” signature only.

Particles collected from location “c” in ISO 7 and “d” in ISO 5 were analyzed in detail due to the high human traffic as well as more activities in assembling flight hardware. The elemental compositions of 25 out of 70 individual particles captured in the ISO 7 location “c” were displayed in Fig. S2. In this location, aggregated particles showed different levels of element compositions, especially carbon signature (Fig. S2Bb–d,b’–d’). The elemental composition weight percentages of the ISO 7 particles showed that particles have little association with elemental carbon and more association with carbonaceous element, suggesting that these inert particles might have originated from organic matter. On the other hand, ISO 5 particles showed that 80% of particles were dominated by elemental carbon and oxygen (Fig. S3). Together with the elemental composition, the morphological features observed in Fig. S3 confirmed that these images were indeed showing biological particles. Subsequently, several individual and aggregated biological particles morphologically resembled biological structures such as fungal spore, hyphae, and pollen, with sizes ranging from 1–50 μm and composed of C and O signatures (Fig. 4).

### Visualization of microbial cell associated with particles using cEFM-SEM-EDXA

**Figure 5:**
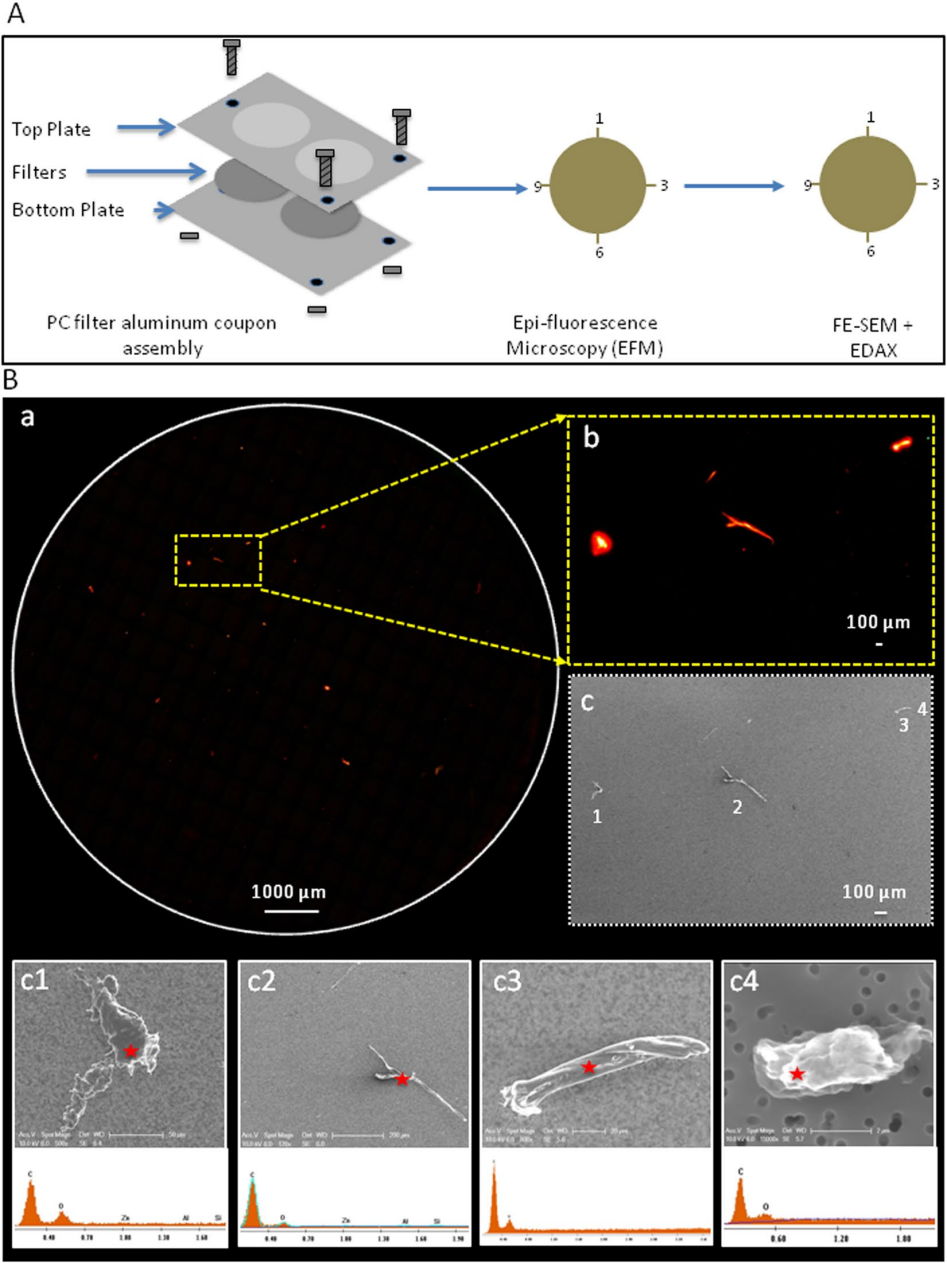
Visualization of fallout particles using cEFM-SEM-EDXA. (**A**) Schematic illustration depicting the procedure for observing fallout particle on polycarbonate filter^50^ from JPL using cEFM-SEM-EDXA. Left side of the illustration shows the aluminum coupon components employed in assembling the PC filter used throughout this study. The coupons were assembled with sterile PC filter in aseptic condition and were deployed in JPL-cleanroom for two weeks for four consecutive sampling events. After two weeks, the coupons were collected and processed for further analysis. The middle image shows how the PC filter is aseptically removed from the coupon, stained with SYTO-9 and PI, and imaged by EFM. After the completion of global imaging, the PC filter was removed and aseptically placed over the coupon with clock like markings to allow for allocation and coordination of observed regions by EFM. Particles were carbon coated and subsequently analyzed by SEM (Right side). (**B**) Correlative microscopy analysis of fallout particles from JPL-cleanroom: (a) A typical global PC filter fluorescence image of fallout particles; (b) Fluorescence image of the field highlighted yellow broken line in (a); (c) SEM image (x35) of a carbon coated region shown in (b); (c1 – c4) SEM images of the field shown in (c) and its corresponding elemental composition.

To determine whether the EFM images were biological or inert in nature, the EFM filters were directly analyzed using the FESEM/EDXA approach (cEFM-SEM-EDXA); the experimental protocol is shown in Fig. 5A. In this technique, particles collected on PC filters were stained with BacLight, tracked by EFM, and the generated image was processed using ImageJ (Fig. 5Ba,b). Subsequently, the PC filters were carbon coated, and the corresponding regions were acquired using FESEM (Fig. 5Bc). This analysis revealed a substantial correlation between the particles observed by EFM and FESEM (Fig. 5Bb,c1–c4). The observed images suggested that the filter was not contaminated and that the particles were not moved during the sample processing and transport. In addition, the detailed chemical compositions of individual particles analyzed showed that they were carbon and oxygen rich, suggesting that all the particles were biogenic in nature, such as fungal hyphae or bacterial spores (Fig. 5Bc2–c4).

**Figure 6:**
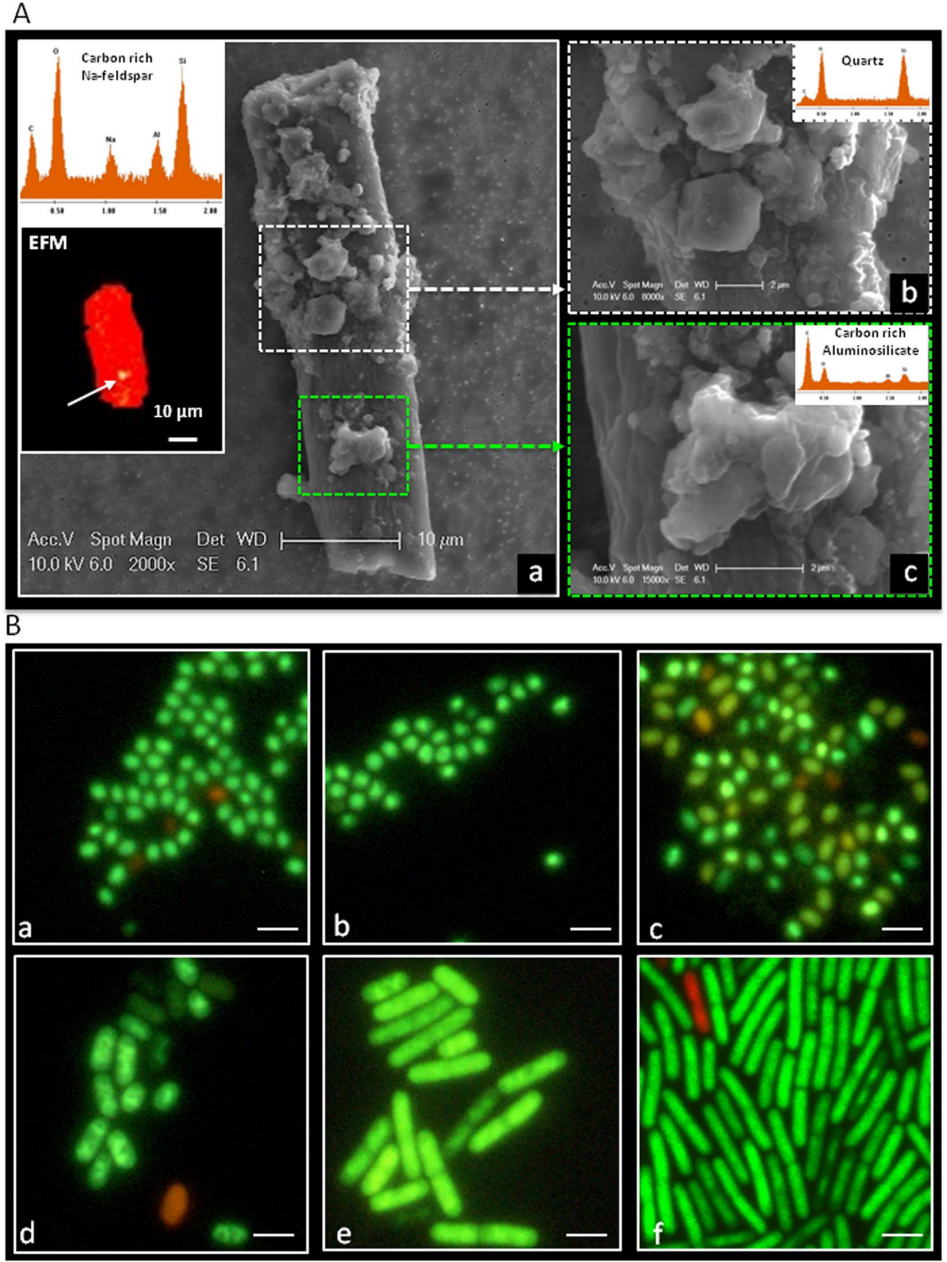
Visualization of biological and inert particle using cEFM-SEM-EDAX and spore revival by time course microscopy. (**A**) A typical SEM image (x2000) of a tubular structured NA-feldspar with aggregates of an irregularly shaped carbon and silica rich particles. Insert top shown is the whole elemental composition of the particles from (a) and the bottom shown is a fluorescence image of the same region from (a); the arrow highlighted that the particles were stained by both SYTO-9 and PI likely to be a biological particle associated with an inert dust particle. (b) SEM image (x8000) of irregular quartz particles on the top of tubular structure shown in (**A**) white broken line boxed region and insert is an elemental composition of the region. (b) SEM image (x15000) smooth rod and irregular shaped on NA-feldspar tubular highlighted green broken boxed region in (a) and the insert is an elemental composition of the selected region. (**B**) Time course epi-fluorescence microscopy of BacLight stained *Bacillus pumillus* SAFR032 spore revival on PC filter incubated with TSA plate for 180 mins at 30 °C.

In some red EFM images, yellow-stained particles were noticed (Fig. 6Aa-white arrow); to identify their biological state, FESEM/EDX analyses were performed. The EDXA-based elemental composition of the red image identified it as carbon rich Na-feldspar, a tectosilicate mineral (Fig. 6Aa). However, EDXA spectra of the yellow-stained material confirmed it as biological material since it was rich in carbon substrate (Fig. 6Ac, insert). In contrast, the red-stained structure possessed quartz signature, a non-biological material (Fig. 6Ab, insert). Frequently, live microbial cells were embedded within the dead biological or inert particles as shown in the Fig. S4. This microscopy approach is a powerful tool in differentiating dead from live biological particles in extremely clean low biomass environments.

### Confirmation of biological materials associated with fallout particles

The cultivability of the biological materials associated with the EFM images (green spots) was examined by growing them using a time-course microscopy technique. Spores of a cleanroom isolate, *Bacillus pumillus* SAFR032, were spotted onto the PC filter, and the spore germination was tracked using BacLight staining. The spores on top of the PC filters were revived when placed onto a TSA agar plate and germinated normally (Fig. 6B). The spore ripening period required 60 min of incubation (Fig. 6Ba–c). After 90 mins of incubation, the elongated cell formation was observed, and fully formed vegetative cells could be observed after 180 mins of incubation (Fig. 6Bd–f). Two PC filters from each location that collected cleanroom particles were subjected to microscopy and culture techniques. The EFM images (Figs S5 and S6A) taken immediately after collection (at 0 hr and 3 hrs incubation; Fig. S5A,B) showed no appreciable live (green) cells, whereas images after 16 hrs of incubation on R2A pre-enrichment showed growth of microbial cells (visible green color via EFM; Fig. S5C). This might be attributed to the transfer of nutrients through the pores of PC filters, stimulating the growth of the microbial particles as shown in Figs S5C and S6A. The microorganisms (*n* = 10) associated with these particles were isolated from the R2A agar plate, purified, and sequenced for their 16S ribosomal gene for the phylogenetic affiliation. The sequencing results of this study confirmed the identity of the spore-forming bacteria as *Bacillus zhangzhouensis* and *Bacillus horneckii* (Fig. S6B)^37^. The results of this study revealed that biological particles existed in aggregate or as free-living in the particles of the spacecraft assembly cleanroom.

### Microorganism and particle association distribution

**Figure 7:**
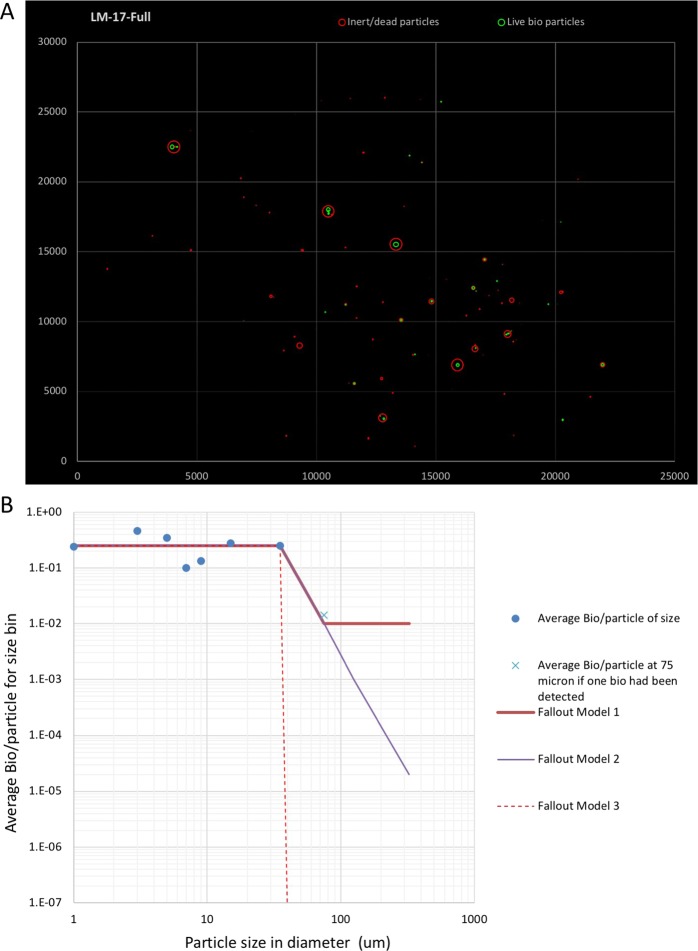
Total viable organisms per particles distribution in a cleanroom facility with three extrapolation methods: (**A**) An example graph of an ImageJ particle count outputs plotted on the of x and y coordinates and over-layered according to its size. (**B**) Fallout Model 1 - the extrapolated trend if one microorganism was detected in the 50–99.9 micron particle bin range and maintained that ratio of bio/particles; Fallout Model 2 - the extrapolated trend maintaining the same slope if one microorganism was detected in the 50–99.9 micron particle bin; and Fallout Model 3 – no microorganisms were detected above 50 microns, and so the trend drops to zero. This implies that microorganisms do not associate with particles larger than 50 μm in a cleanroom environment.

Based on ImageJ analyses, the diameter size of the cleanroom particles ranged from 0.5 μm to 500 μm (Supplementary Datafile Tables S1 and S2). The combined analyses of EFM and FESEM/EDXA enabled the study to group the particle size and determine the association of microbial cells (Fig. 7). Both microbial cells (C signature in EDXA) and inert particles were accounted in this analysis. In both facilities, the majority of the larger particles (20–50 µm) had microorganisms. Furthermore, only ~5.2% of the smaller particles (0–1.9 µm) were free-floating and exhibited green fluorescence (EFM; BacLight staining), confirming that they were microbial cells. A third set of particles binned into the larger category consisted of the size range >50 µm. From this analysis, three distinct models were extrapolated (Fig. 7). They are: Fallout Model 3 – this model reflects the actual as-measured data. There were no biological particles observed over 50 microns using both the FEM and FESEM methods, as represented by the dotted line. This implies that microorganisms do not associate with particles larger than 50 μm in a cleanroom environment. On the other hand, if we were to find one biological particle greater than 50 µm, and maintained the resulting slope, the extrapolated line is captured by Fallout Model 2. If, however, the slope was maintained at a constant value, the resulting extrapolated model is captured in Fallout Model 1. The purpose for the three extrapolated fallout models is to inform the bounds of the overall spacecraft-level particle and biological transport model. These conservative and nominal estimates allow us to understand the sensitivities of the estimates in order to effectively quantify the cleanliness of the acquired samples of unknown origin.

## Discussion

One of the main objectives for NASA’s current and future missions is to prevent forward and backward contamination^10,38^. The stringent maintenance of NASA cleanroom surfaces and air kept microbial counts extremely low which enable in preventing hitchhikers associated with spacecraft surfaces^6,14,15^. A real-time quantification of bioaerosols in JPL cleanrooms using BioVigilant IMD-A 350 system (Azbil Corporation, Tucson AZ, USA) showed a positive correlation between human presence and elevated bioaerosol counts. Since the bioaerosol presence could not be confirmed using microbiological and DNA-based methods for the presence of microorganisms, visualization using correlative microscopy methods performed during this study should be considered^39^. The goal of this study is to determine the association of microorganisms with the inert, natural cleanroom fallout particles and to ascertain whether microorganisms prefer to adhere to a particular particle size. A novel microscopy technique was developed, and by utilizing various molecular techniques, particles and associated microbial phylogeny were characterized. By providing an accurate and confident assessment of the microbes associated with cleanroom particles, it is possible to protect the health of the people who occupy the room for long duration in assembling critical components. The outcome from this study would help to address the distribution of microbes to particles on the spacecraft and the associated environment, as well as help other pharmaceutical and medical industries. Multiple studies have used SYTO9 and PI for the estimation of live and dead biological particles and electron microscopy to characterize morphology and elemental composition; however, no report is available where all of these techniques were used together^40^. Here, for the first time, we designed and implemented live/dead staining combined with correlative epifluorescence, electron microscopy (c-EFM-SEM-EDXA), and DNA sequencing technology to characterize live microbial particles isolated from the JPL-related spacecraft assembly facilities.

In general, airborne microbes can be associated with inert particles of various morphology and sizes^22,23^. Previous studies have shown microbial ecology and diversity in spacecraft assembly facility cleanrooms utilizing traditional and molecular microbiology methods; however, those techniques were for characterizing molecular microbial community analyses in bulk samples collected from surfaces, but none of them characterized individual particles^7,10,13,14,21,23,41–44^. The particles characterized in this study utilizing microscopy techniques ranged from 0.5–500 μm in diameter and displayed morphologies that varied from carbonaceous tubular structures to regular- and irregular-shaped biological and inert particles. Additionally, EDX analyses of the large inert particles (10–500 μm in diameter) showed that Al and Si groups were dominated and that the shapes of particles varied from triangular to irregular. Notably, fiber-like individual carbonaceous inert particles were dominated by tubular structures ranging from 50–200 μm in diameter. Interestingly, biological particles were associated with sizes ranging from 1–50 μm. A spherical or tubular-shape biological particle, such as bacteria, fungal spore, and hyphae, were also observed with C and O signatures as well as minor amounts of other essential elements (Na, Mg, K, Ca, and Cl). Furthermore, soil-derived natural fallout SiO_2_ particles were also found in the cleanroom with other elements such as Al, Mg, and Na.

The standard particle characterization method, using both light and electron microscopy methods, was useful in characterizing the particles, but incapable of distinguishing between biological and inert particles^25,45^. Recent developments on advanced correlative microscopy have created a powerful biological research tool to identify specific particles (either live or dead) that have been pre-selected using light microscopy in conjunction with SYTO9 and PI staining prior to imaging the same particles by electron microscopy. Results from correlative microscopy in this study suggested that samples have not cross-contaminated or particle removed during sample preparation, processing, transporting, and imaging. Furthermore, the characterization of such samples contributed significantly to understanding the morphological characterization of cleanroom fallout particles.

This study’s isolation of bacterial endospores belonging to the members of *Firmicutes* were also reported to be the most prominent groups of bacteria detected in NASA cleanroom and assembly facility associated surfaces^14,15,21,44^. Furthermore, the dominant fallout *Bacillus* spore-forming particles found in this study are considered likely to impact spacecraft, thus posing the greatest risk of forward contamination^6,46^. It is also reported that endospore-forming bacteria as noticed in this study would be the most possible survivors of spacecraft disinfection^47^. The isolation of *B. zhangzhouensis*, which falls into the same phylogenetic clade as that of *B. pumilus*, an extraordinary UV-resistant spore-forming bacteria^48^, begs the question about the UV-resistant characteristics of the spores associated with the particles in this study. In addition, the isolation of *B. hornekiae*, a space vacuum surviving endospore-former^49^, from the particles in this study indicates that it should be characterized for various decontamination and sterilization regimes. The live biological fallout particles as observed via microscopy were cultured on the specific media needed to perform downstream DNA sequencing for the species level identity. These particle-associated biological materials were archived for further analyses, which would enable us to understand their resistance characteristics toward various cleaning and sterilization techniques.

In conclusion, correlative epifluorescence and electron microscopy coupled with energy dispersive x-ray analysis (cEFM-SEM-EDX) and ImageJ processing are useful for the evaluation of fallout particles and their corresponding size distribution. The proposed method is precise and rapid. Furthermore, the recommended method could be very useful for investigating the behavior of the particles themselves, according to their morphology and size, on the spacecraft assembly cleanroom facilities.

## Supplementary information

Supplementary Dataset 1

## References

[1] Pecault IT, Godefroy P, Escoubas L (2017). Qualification testing of an innovative system for monitoring particle contamination fallout. Sensor. Actuat. A: Phys..

[2] Peters S (1995). Particle Fallout in a Class 100,000 High-Bay Aerospace Cleanroom. Journal of the IES.

[3] Favero MS, Puleo JR, Marshall JH, Oxborrow GS (1966). Comparative levels and types of microbial contamination detected in industrial clean rooms. Appl. Microbiol..

[4] Favero MS, Puleo JR, Marshall JH, Oxborrow GS (1968). Comparison of microbial contamination levels among hospital operating rooms and industrial clean rooms. Appl. Microbiol..

[5] FED-STD-209. (ed. U.S. General Services Administration) (General Services Administration, Washington DC, 1992).

[6] La Duc MT (2007). Isolation and characterization of bacteria capable of tolerating the extreme conditions of clean room environments. Appl Environ Microbiol.

[7] Venkateswaran K (2001). Molecular microbial diversity of a spacecraft assembly facility. Syst Appl Microbiol.

[8] Hauenstein, L. R. In *Particles in Gases and Liquids 3: Detection, Characterization, and Control* (ed. Mittal, K. L.) 189–201 (Springer US, 1993).

[9] Licina D (2016). Concentrations and Sources of Airborne Particles in a Neonatal Intensive Care Unit. PLoS One.

[10] Bruckner, J., Osman, S., Conley, C., Venkateswaran, K. & Schaechter, M. Space microbiology: planetary protection, burden, diversity and significance of spacecraft associated microbes. *Encyclopedia of Microbiology, edited by* Schaechter, M.*, Elsevier, Oxford*, 52–65 (2008).

[11] COSPAR. (COSPAR, Houston, TX, 2002).

[12] NASA. (National Aeronautics and Space Administration, Washington, D.C., 2005).

[13] Puleo JR (1977). Microbiological profiles of the Viking spacecraft. Appl. Environ. Microbiol..

[14] La Duc MT, Kern RG, Venkateswaran K (2004). Microbial monitoring of spacecraft and associated environments. Microb. Ecol..

[15] La Duc MT, Nicholson W, Kern R, Venkateswaran K (2003). Microbial characterization of the Mars Odyssey spacecraft and its encapsulation facility. Environ. Microbiol..

[16] La Duc MT, Vaishampayan P, Nilsson HR, Torok T, Venkateswaran K (2012). Pyrosequencing-derived bacterial, archaeal, and fungal diversity of spacecraft hardware destined for Mars. Appl. Environ. Microbiol..

[17] Vaishampayan P, Osman S, Andersen G, Venkateswaran K (2010). High-density 16S microarray and clone library-based microbial community composition of the Phoenix spacecraft assembly clean room. Astrobiology.

[18] Minich, J. J. *et al*. KatharoSeq Enables High-Throughput Microbiome Analysis from Low-Biomass Samples. *mSystems* **3**, 10.1128/mSystems.00218-17 (2018). 10.1128/mSystems.00218-17 PMC5864415 29577086

[19] Cooper M (2011). Comparison of innovative molecular approaches and standard spore assays for assessment of surface cleanliness. Appl Environ Microbiol.

[20] Lin, Y. In 2012 I*EE*E Aero*space Conference*. 1–7.

[21] Checinska A (2015). Microbiomes of the dust particles collected from the International Space Station and Spacecraft Assembly Facilities. Microbiome.

[22] Pachauri T, Singla V, Satsangi A, Lakhani A, Kumari KM (2013). SEM-EDX characterization of individual coarse particles in Agra, India. Aerosol and Air Quality Research.

[23] Yamaguchi N, Ichijo T, Sakotani A, Baba T, Nasu M (2012). Global dispersion of bacterial cells on Asian dust. Sci. Rep..

[24] Coccia AM, Gucci PM, Lacchetti I, Paradiso R, Scaini F (2010). Airborne microorganisms associated with waste management and recovery: biomonitoring methodologies. Ann. Ist. Super. Sanita.

[25] Dubey GP (2016). Architecture and Characteristics of Bacterial Nanotubes. Dev. Cell.

[26] Vaishampayan P (2013). New perspectives on viable microbial communities in low-biomass cleanroom environments. ISME J.

[27] Moissl C, Bruckner JC, Venkateswaran K (2008). Archaeal diversity analysis of spacecraft assembly clean rooms. ISME J.

[28] Moissl C (2007). Molecular bacterial community analysis of clean rooms where spacecraft are assembled. Fems Microbiol Ecol.

[29] Malli Mohan, G., Benardini, J., Hendrickson, R., Venkateswaran, K. & Stricker, M. Characterization of Biological Fallout Particles of Cleanrooms to Measure Spacecraft Cleanliness. *47th International Conference on Environmental Systems, Conference Proceedings*, https://ttu-ir.tdl.org/handle/2346/72973 (2017).

[30] Schneider CA, Rasband WS, Eliceiri KW (2012). NIH Image to ImageJ: 25 years of image analysis. Nat. Methods.

[31] Harrold ZR, Hertel MR, Gorman-Lewis D (2011). Optimizing Bacillus subtilis spore isolation and quantifying spore harvest purity. J Microbiol Methods.

[32] Pandey R (2013). Live Cell Imaging of Germination and Outgrowth of Individual Bacillus subtilis Spores; the Effect of Heat Stress Quantitatively Analyzed with SporeTracker. PLoS One.

[33] Lederberg J, Lederberg EM (1952). Replica plating and indirect selection of bacterial mutants. J. Bacteriol..

[34] Lane, D. J. In *Nucleic Acid Techniques in Bacterial Systematics* Vol. 1 (eds Stackebrandt, E. & Goodfellow, M.) 115–175 (Wiley, 1991).

[35] Turner S, Pryer KM, Miao VP, Palmer JD (1999). Investigating deep phylogenetic relationships among cyanobacteria and plastids by small subunit rRNA sequence analysis. J. Eukaryot. Microbiol..

[36] Kwan K (2011). Evaluation of Procedures for the Collection, Processing, and Analysis of Biomolecules from Low-Biomass Surfaces. Applied and Environmental Microbiology.

[37] Vaishampayan P (2010). *Bacillus horneckiae* sp. nov., isolated from a spacecraft-assembly clean room. Int. J. Syst. Evol. Microbiol..

[38] NRC. (National Research Council. National Academies Press, Washington, D.C., 2006).

[39] Cruz, S. *et al*. Real-time Quantification of Size-resolved Bioaerosols and Inert Particles In Spacecraft Assembly Cleanrooms. *42nd COSPAR Scientific Assembly* **42** (2018).

[40] Hara K, Zhang D (2012). Bacterial abundance and viability in long-range transported dust. Atmos. Environ..

[41] Pierson DL (2001). Microbial contamination of spacecraft. Gravit. Space Biol. Bull..

[42] Puleo JR, Fields ND, Moore B, Graves RC (1970). Microbial contamination associated with the Apollo 6 spacecraft during final assembly and testing. Space Life Sci..

[43] Puleo JR, Oxborrow GS, Fields ND, Herring CM, Smith LS (1973). Microbiological profiles of four Apollo spacecraft. Appl. Microbiol..

[44] Satomi M, La Duc MT, Venkateswaran K (2006). *Bacillus safensis* sp. nov., isolated from spacecraft and assembly-facility surfaces. Int. J. Syst. Evol. Microbiol..

[45] Loussert Fonta C, Humbel BM (2015). Correlative microscopy. Arch. Biochem. Biophys..

[46] Schuerger AC, Mancinelli RL, Kern RG, Rothschild LJ, McKay CP (2003). Survival of endospores of *Bacillus subtilis* on spacecraft surfaces under simulated Martian environments: implications for the forward contamination of Mars. Icarus.

[47] Sagripanti JL, Bonifacino A (1999). Bacterial spores survive treatment with commercial sterilants and disinfectants. Appl. Environ. Microbiol..

[48] Vaishampayan P, Rabbow E, Horneck G, Venkateswaran K (2012). Survival of *Bacillus pumilus* spores for a prolonged period of time in real space conditions. Astrobiology.

[49] Krebs, J. E. *et al*. Assessment of *Bacillus horneckiae* spore survival under simulated space ISS conditions. 2012 *American Society for Microbiology General Meeting*, *San Francisco Convention Center, San Francisco, CA* (2012).

[50] Weller R, Bateson MM, Heimbuch BK, Kopczynski ED, Ward DM (1992). Uncultivated cyanobacteria, Chloroflexus-like inhabitants, and spirochete-like inhabitants of a hot spring microbial mat. Appl. Environ. Microbiol..

