# Supplementary material for: Microscopic Characterization of Biological and Inert Particles Associated with Spacecraft Assembly Cleanroom
Source: Sci Rep. 2019 Oct 3;9:14251. doi: 10.1038/s41598-019-50782-0 (PMC6776515; doi:10.1038/s41598-019-50782-0)
Supplement: Supplementary file 1 — Supplementary Dataset 1 [file 41598_2019_50782_MOESM1_ESM.pdf]

Microscopic Characterization of Biological and Inert Particles Associated with Spacecraft Assembly Cleanroom Particles

Ganesh Babu Malli Mohan[1#], Moogega Cooper Stricker[1#], and Kasthuri Venkateswaran[1*]

[1]Jet Propulsion Laboratory, California Institute of Technology,
Biotechnology and Planetary Protection Group, Pasadena, CA 91109

[#]Equally Contributed
*Corresponding author
California Institute of Technology, Jet Propulsion Laboratory
Biotechnology and Planetary Protection Group;
4800 Oak Grove Dr., Pasadena, CA 91109
Tel: (818) 393-7795; Fax: (818) 3934176
E-mail: kjvenkat@jpl.nasa.gov

Figure S1

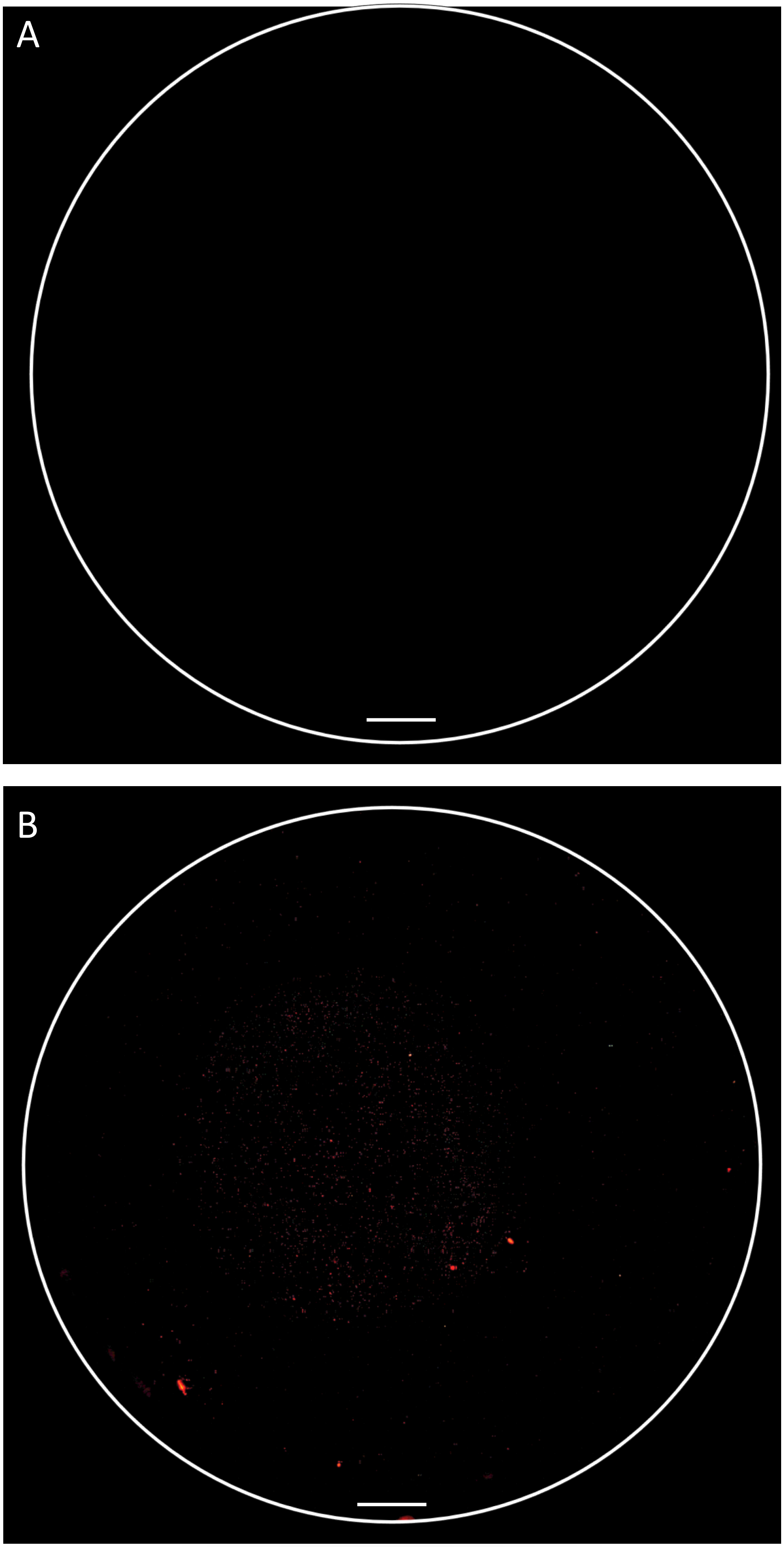

Figure S2

A

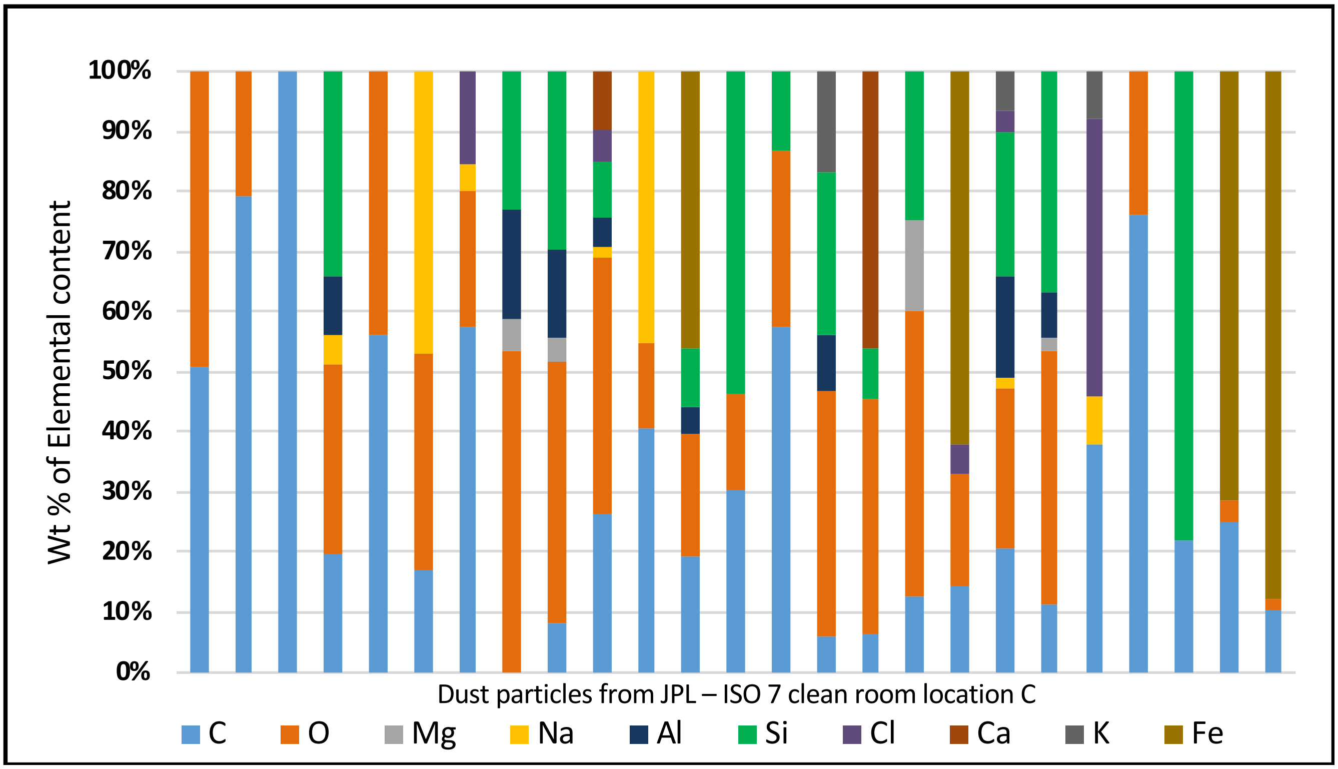
100%
90%
80%
70%
60%
50%
40%
30%
20%
10%
0%
Wt % of Elemental content
Dust particles from JPL – ISO 7 clean room location C
C
O
Mg
Na
Al
Si
Cl
Ca
K
Fe

B

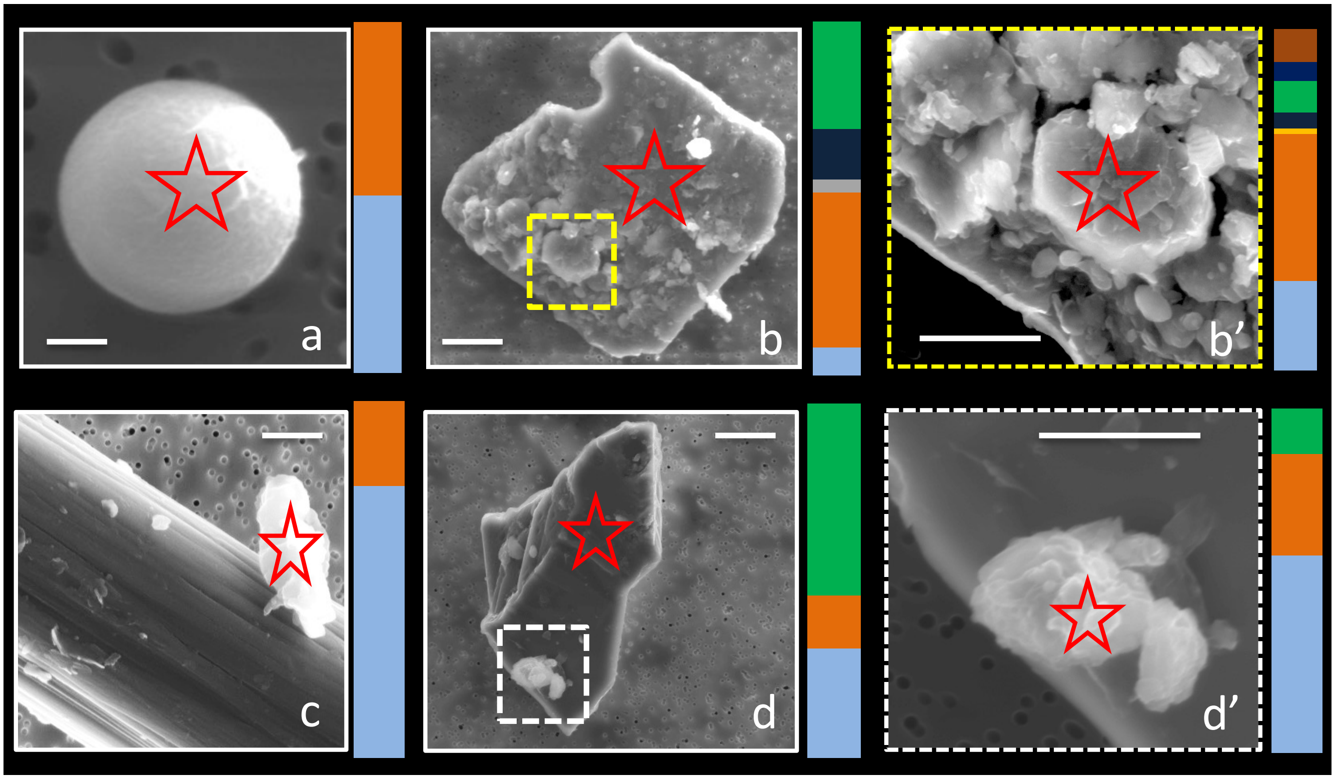
a
b
b'
c
d
d'

Figure S3

A

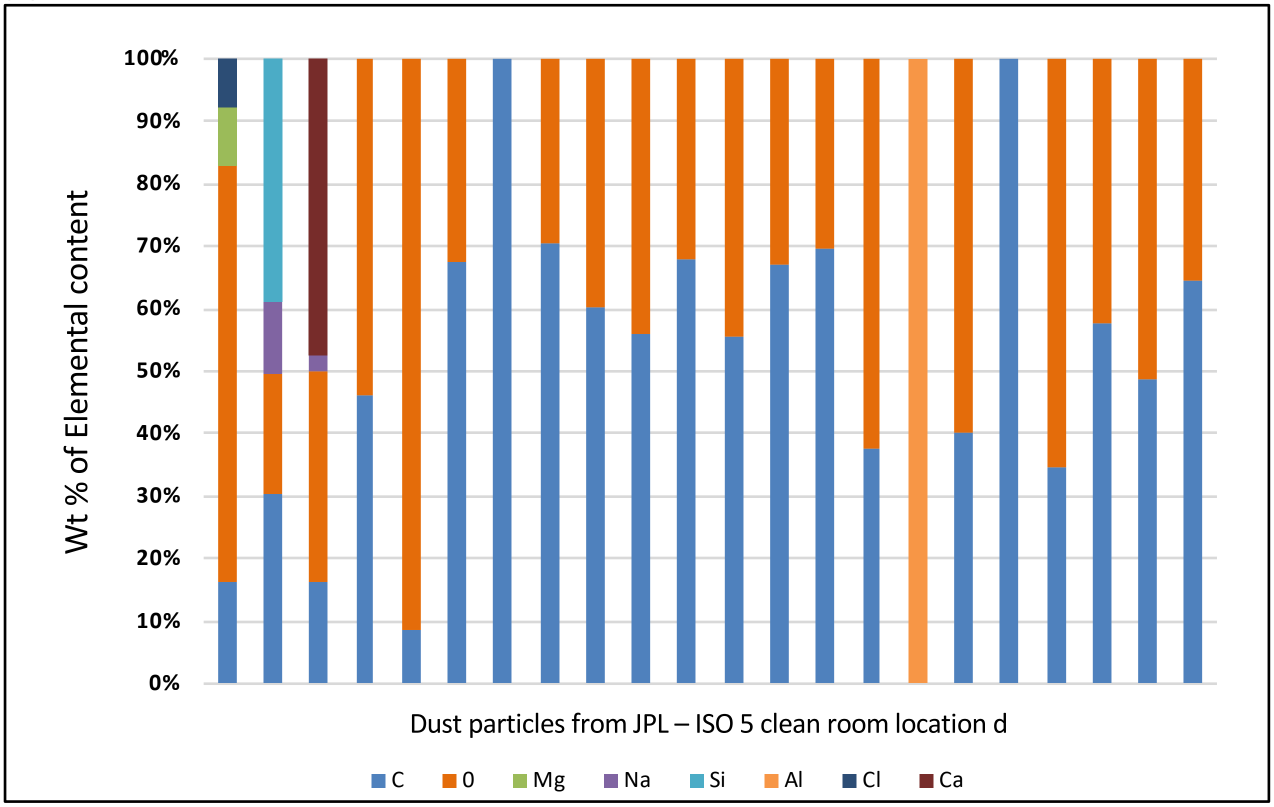

100%
90%
80%
70%
60%
50%
40%
30%
20%
10%
0%
Wt % of Elemental content
Dust particles from JPL – ISO 5 clean room location d
C
0
Mg
Na
Si
Al
Cl
Ca

B

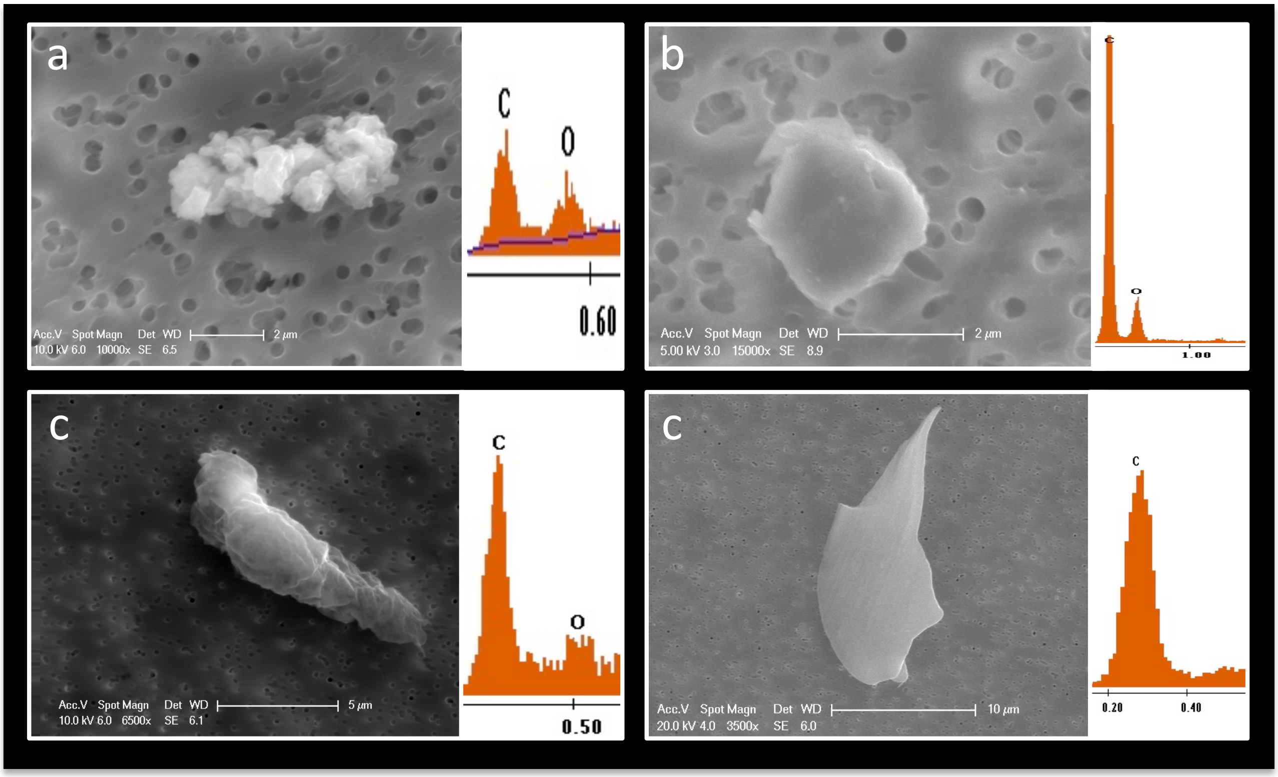

a
C
O
Acc.V Spot Magn Det WD 2 µm
10.0 kV 6.0 10000x SE 6.5
0.60
b
c
o
Acc.V Spot Magn Det WD 2 µm
5.00 kV 3.0 15000x SE 8.9
1.00
c
C
O
Acc.V Spot Magn Det WD 5 µm
10.0 kV 6.0 6500x SE 6.1
0.50
c
C
Acc.V Spot Magn Det WD 10 µm
20.0 kV 4.0 3500x SE 6.0
0.20
0.40

Figure S4

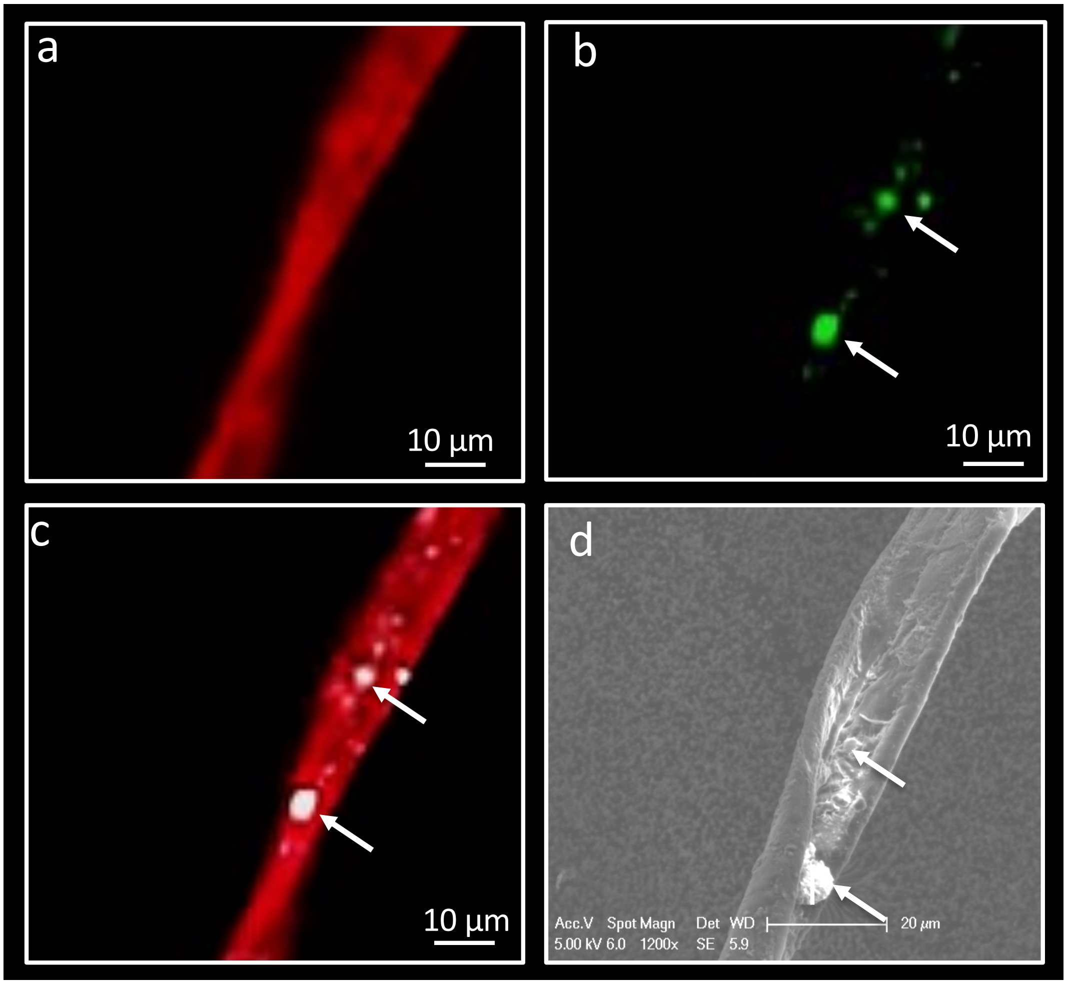
a
10 µm
b
10 µm
c
10 µm
d
Acc.V Spot Magn Det WD
5.00 kV 6.0 1200x SE 5.9
20 µm

Figure S5

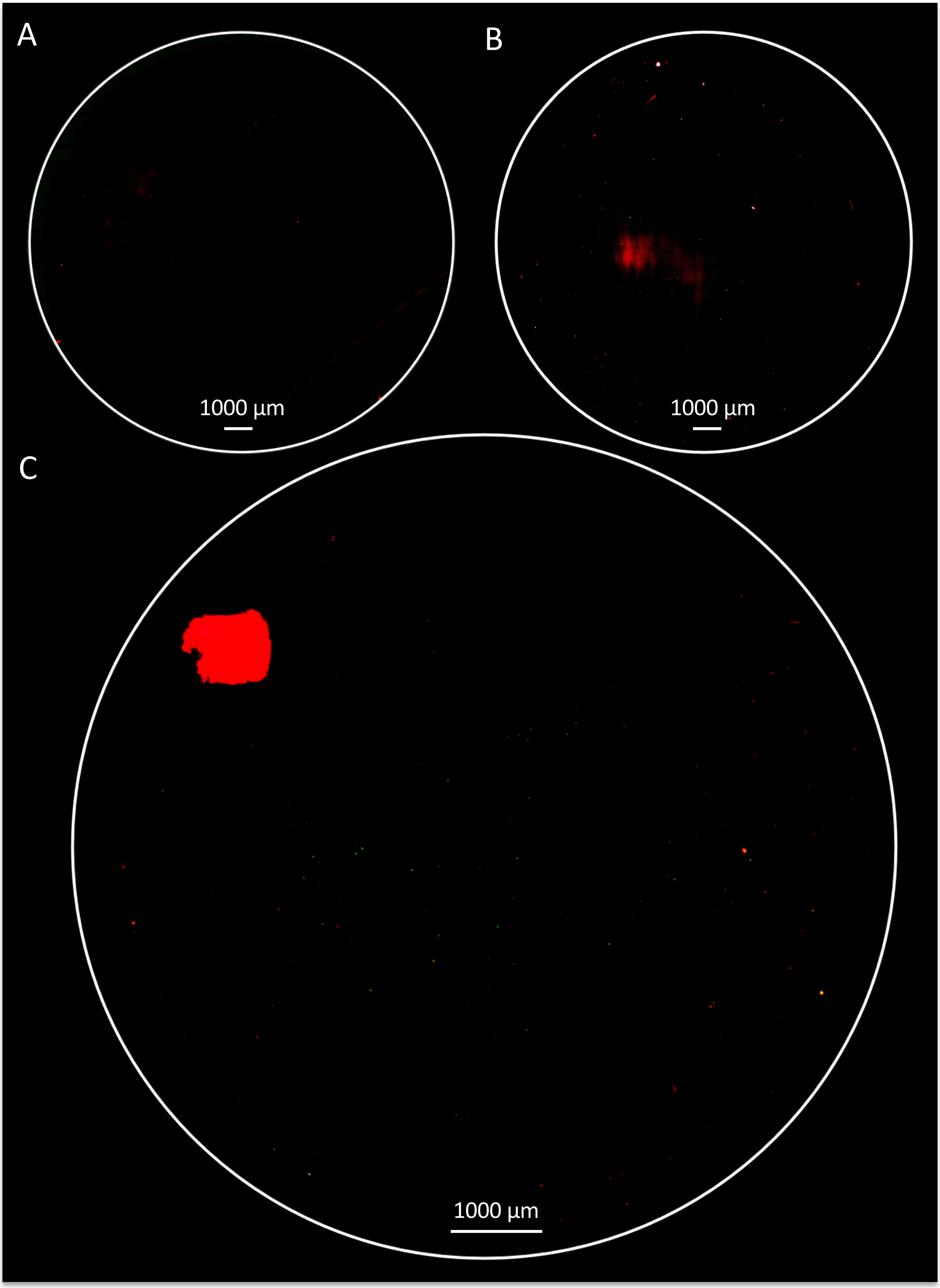

Figure S6

A

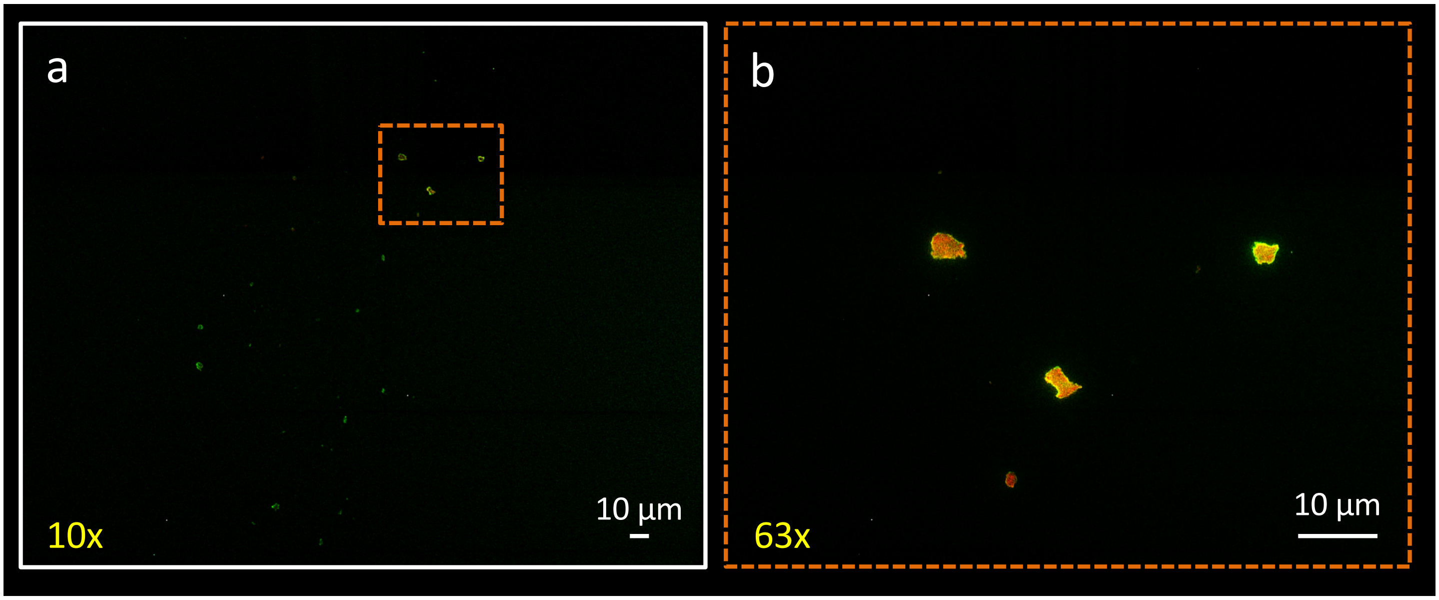

a
10x
10 μm
b
63x
10 μm

B

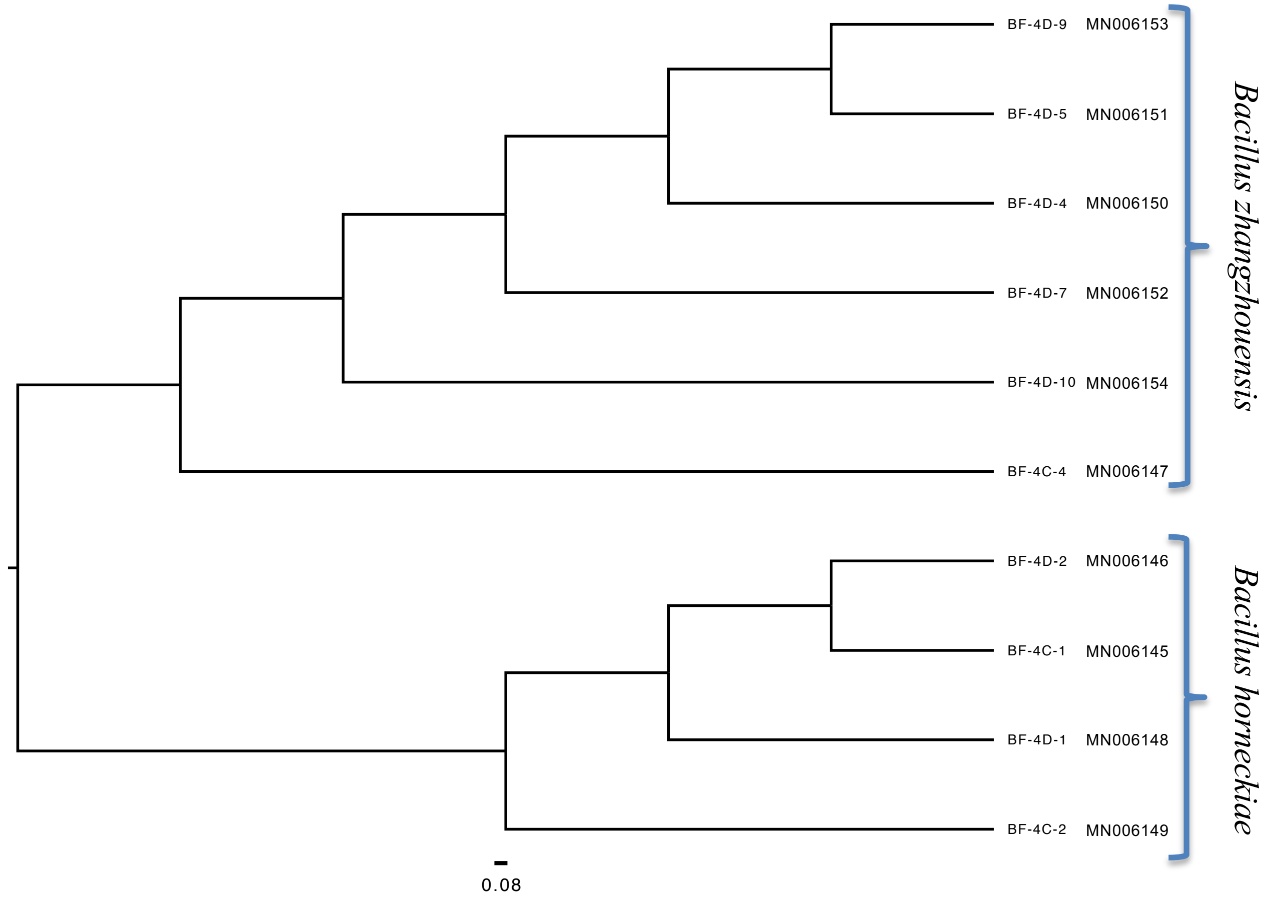

BF-4D-9 MN006153
BF-4D-5 MN006151
BF-4D-4 MN006150
BF-4D-7 MN006152
BF-4D-10 MN006154
BF-4C-4 MN006147
BF-4D-2 MN006146
BF-4C-1 MN006145
BF-4D-1 MN006148
BF-4C-2 MN006149
Bacillus zhangzhouensis
Bacillus horneckiae
0.08

Figure S1. Epi-fluorescence imaging of polycarbonate filters
(A) A typical epi-fluorescence image of the whole sterile control PC filter stained with SYTO-9 and PI
(B) A typical epi-fluorescence image of the whole PC filter trapped with Model Microbial Community (MMC) stained with SYTO-9 and PI

Figure S2. EDXA spectral analysis of fallout particles collected from JPL-SAF
(A) Bar diagram represents the elemental composition of 25 selected fallout particles from JPL-SAF.
(B) Selected SEM image from the from (A): (a) a typical carbon ash with spherical shape; (b) irregular shaped magnesium aluminosilicate with carbonaceous aggregate particles; (b') SEM image (x12000) of carbonaceous aggregate on magnesium aluminosilicate yellow broken boxed region shown in (b); (c) a carbonaceous rod shaped likely to be a biological particle attached to the carbonaceous fiber; (d) rough rod shaped bacteria-like particle associated with irregular shaped silica particle [54] SEM image (x15000) of rough rod shaped bacteria like particle with higher carbon signature shown broken white boxed region when compare with (d).

Figure S3. EDXA spectral analysis of fallout particles collected from JPL-DS-233
(A) Bar diagram represents the elemental composition of 22 selected fallout particles from JPL-DS-233.
(B) Selected SEM image from (A): (a) a typical irregular rod shaped carbonaceous particle, likely to be a biological particle; (b-c) carbonaceous particle with irregular morphology

Figure S4. cEFM-SEM
A typical fluorescence image of selected area shows PI signal from fungal hyphae like structure (a), SYTO9 signal from a live microbial particle (b) and an composite image of (a) and (b) (c). SEM image (x1200) of a tubular structured carbon and oxygen rich particles (d) image. White arrow indicates the precise correlation of the live microbial particle by both microscope technique.

Figure S5. Microbial growth experiment and epi-fluorescence microscopy analysis
Epi-fluorescence images of fallout particles PC filter collected from JPL-DS-233 cleanroom and placed over R2A plate for growth experiment. (A) Fluorescence image of whole PC filter immediately after it was collected from the cleanroom (0 hour); (B) Fluorescence image of whole PC filter after 3 hrs of incubation on R2A plate; and (C) Fluorescence image of the whole PC filter after 16 hours of incubation on R2A plate.

Figure S6. Epi-fluorescence imaging of fallout particle growth experiment
(A) Different magnification of same field of fluorescence image of the fallout particles of the selected area from global PC filter collected from JPL-SAF cleanroom (a-b).
(B) The phylogenetic tree shows bacterial species isolated from fallout coupon collected in JPL cleanroom. The name of the strains designated are as BF4C/D-1; BF – Biological fallout; 4C/D – fourth deployment location "C" SAF/"D" DS; 1 – number of isolate identified.
